# Memorial Oration in Honor of Ephraim McDowell

**Published:** 1880-01

**Authors:** 


MEMORIAL ORATION IN HONOR OF EPHRAIM MCDOWELL, ("the Father of Ovariotomy," by Samuel D. Gross, M.D,, LL.D., D.C.L., Oxon. Delivered at Danville, Ky., at the dedication of the Monument erected to the memory of Dr. Ephraim McDowell by the Kentucky State Medical Society, May 14th, 1879. Published by the Society. 1879.

This oration is in the usual happy style of its eminent author, and is well adapted to the occasion for which it was intended. The event of its presentation is an epoch that will never be forgotten.

